# ESKAPE pathogen incidence and antibiotic resistance in patients with bloodstream infections at a referral hospital in Limpopo, South Africa, 2014–2019: A cross-sectional study

**DOI:** 10.4102/ajlm.v13i1.2519

**Published:** 2024-11-29

**Authors:** Tiyani C. Mthombeni, Johanita R. Burger, Martha S. Lubbe, Marlene Julyan, Molebogeng R. Lekalakala-Mokaba

**Affiliations:** 1 Department of Medicine Usage in South Africa (MUSA), Faculty of Health Sciences, North-West University, Potchefstroom, South Africa; 2 Division of Medical Microbiology, Department of Pathology, Faculty of Health Sciences, University of Limpopo, Polokwane, South Africa; 3 Polokwane Laboratory, National Health Laboratory Service, Polokwane, South Africa

**Keywords:** *Acinetobacter baumannii*, bloodstream infections, antibiotic resistance, *Klebsiella pneumoniae*, South Africa, *Staphylococcus aureus*

## Abstract

**Background:**

There is a paucity of research on the incidence and antimicrobial resistance (AMR) of *Enterococcus faecium, Staphylococcus aureus, Klebsiella pneumoniae, Acinetobacter baumannii, Pseudomonas aeruginosa*, and *Enterobacter* spp. (ESKAPE) pathogens in Africa because of the inadequate establishment of AMR surveillance systems.

**Objective:**

This study reports on the incidence and AMR of bloodstream ESKAPE pathogens at a referral hospital in northern South Africa.

**Methods:**

This retrospective descriptive study used routinely collected bloodstream isolates (pathogen identification and antimicrobial susceptibility testing performed using automated systems) from the South African National Health Laboratory Service, from January 2014 to December 2019. Resistant phenotypes analysed included methicillin-resistant *S. aureus* and carbapenem-resistant *A. baumannii*.

**Results:**

The ESKAPE pathogen incidence rate was stable from 2014 to 2019 (*p* = 0.133). The most isolated pathogens were *S. aureus* (268/746; 35.9%) and *A. baumannii* (200/746; 26.8%). *Staphylococcus aureus* increased from 39 isolates in 2014 to 75 in 2019 (*p* = 0.132). The incidence rate of *A. baumannii* increased from 11.9% (16/134) in 2015 to 37.8% (68/180) in 2019 (*p* = 0.009). Most isolates (417/746; 55.9%) were from the neonatal ward. Carbapenem-resistant *A. baumannii* increased from 68.8% (11/16) in 2014 to 75.0% (51/68) in 2019 (*p* = 0.009). Methicillin-resistant *S. aureus* decreased from 56.0% (14/25) in 2016 to 17.3% (13/75) in 2019 (*p* = 0.260).

**Conclusion:**

Routine data provide essential information on the incidence of ESKAPE pathogens and AMR phenotypes, serving as a basis for an antibiogram, a surveillance tool in antibiotic stewardship programmes.

**What this study adds:**

The study provided local information on the incidence and AMR pattern of ESKAPE pathogens, which is essential when developing empiric treatment protocols for appropriate antibiotic prescribing and infection prevention and control practices.

## Introduction

Based on estimates from the Global Burden of Disease study, bacterial infections and antimicrobial resistance (AMR) were respectively responsible for 7.7 million and 4.9 million global deaths in 2019, making them the second leading cause of death after ischaemic heart disease.^1,2^ Therefore, AMR has become a global public health concern.^2,3^ The World Health Organization’s (WHO) African region is no exception. In 2019, this region had an estimated 1.05 million deaths that were associated with AMR.^4^ However, AMR surveillance data in the region are scarce.^2,5^

The inappropriate use of antibiotics is the leading cause of multidrug-resistant pathogens,^3^ particularly in low- and middle-income countries, where multidrug-resistant bacteria accounted for 15.5% of healthcare-acquired infections in 2011.^6^ Most multidrug-resistant healthcare-associated infections are caused by six pathogens, namely *Enterococcus faecium, Staphylococcus aureus, Klebsiella pneumoniae, Acinetobacter baumannii, Pseudomonas aeruginosa*, and *Enterobacter* spp., known as ESKAPE, which are capable of ‘escaping’ the biocidal properties of antibiotics.^6,7^ The ESKAPE pathogens are characterised as opportunistic infections and are associated with the highest risk of mortality and increased healthcare costs.^6,7,8^ In 2017, the WHO released its first edition of the 12 bacterial drug-pathogen priority list – a list of drug-pathogen combination bacteria that pose the greatest threat to human health because of the emergence of multidrug resistance.^9^ All ESKAPE drug-pathogen resistant combinations – for example critical priority carbapenem-resistant *A. baumannii* (CRAB), carbapenem-resistant *P. aeruginosa*, carbapenem-resistant *K. pneumoniae* (*K. pneumoniae*), third-generation cephalosporin-resistant *K. pneumoniae* (3GCR *K. pneumoniae*), high priority vancomycin-resistant *E. faecium*, methicillin-resistant *S. aureus* (MRSA), third-generation cephalosporin- and carbapenem-resistant *Enterobacter* spp., and vancomycin-resistant *S. aureus –* are on the WHO’s priority list and serve as important indicators for AMR surveillance and research.^9^

Bacterial bloodstream, lower-respiratory tract, and intra-abdominal infections dominated the global and regional burdens of AMR in 2019.^1,2,4^ The six ESKAPE pathogens were among the 10 commonly isolated bloodstream infections responsible for AMR-related deaths worldwide in 2019.^1,2^ Low- and middle-income countries, especially in Africa, have a high burden of bloodstream infections by ESKAPE pathogens.^2,4,8,10^ The AMR status of specific healthcare settings varies based on the epidemiology of bacterial infections, the antibiotics used, and the geographic area.^1,2,11^

In South Africa, there were 39 000 AMR-related deaths in 2019.^12^ According to national laboratory surveillance data, 40.0% of positive blood cultures in South Africa contain one or more ESKAPE pathogens.^11^ Between 2014 and 2019, the bloodstream pathogens isolated the most in neonates in South Africa were *K. pneumoniae* (28.0%), *A. baumannii* (14.0%), and *S. aureus* (12.0%).^13^ Similarly, in 2020, from a mixed population of patients, *K. pneumoniae* was the ESKAPE pathogen isolated the most in the bloodstream in South Africa, followed by *S. aureus, A. baumannii, E. faecium* and *P. aeruginosa*.^11^

The susceptibility of *K. pneumoniae* to meropenem in South Africa decreased significantly from 98.0% in 2014 to 89.0% in 2019 (*p* = 0.033). During the same period, the *A. baumannii* isolates decreased susceptibility to meropenem (23.0% to 12.0%; *p* = 0.051).^13^ The latest (2020) AMR data for ESKAPE pathogens in South Africa showed that the incidence of extended-spectrum beta-lactamase-producing *K. pneumoniae* increased from 65.0% in 2016 to 70.0% in 2020, and carbapenem-resistant *K. pneumoniae* was at 40.0%.^11^ Methicillin-resistant *S. aureus* decreased from 23.0% in 2016 to 18.0% in 2020. Also in 2020, CRAB was 80.0%, carbapenem-resistant *P. aeruginosa* was 33.0%, and vancomycin-resistant *E. faecium* was 1.3%, with provincial variations attributed to differences in the empirical use of different antibiotics.^11^

Previously, South Africa conducted sentinel AMR surveillance mainly in urban academic hospitals. Therefore, there was inadequate AMR surveillance data from rural hospitals, particularly those in Limpopo province.^14,15,16,17^ A nationwide public sector study^13^ and several single-centre bloodstream culture studies from Gauteng,^18^ KwaZulu-Natal^19^ and the Western Cape^20,21^ show incidence and AMR variability of ESKAPE pathogens according to the health sector (public versus private), location, hospital ward, patient age (e.g., neonates versus adults), level of healthcare (i.e., district, regional, and national hospitals), the source of infection (e.g., community-acquired versus hospital-acquired infections), and increased antibiotic resistance of Gram-negative bacteria. These observed variations emphasise the importance of local data on the incidence and antibiotic resistance, particularly for local policy and practice interventions such as developing empirical treatment formularies by hospital infection prevention and control and antibiotic stewardship programmes.^11,13^ The surveillance of ESKAPE pathogen incidence and priority drug-pathogen-resistant combinations must therefore be prioritised and strengthened to understand their magnitude.^2,8,10^ Against this background, this study reports on the incidence of ESKAPE pathogens, selected combinations of WHO priority drug-pathogen resistance and resistance to Reserve (last resort) antibiotics using routinely collected data of patients with bloodstream infections Limpopo province, South Africa.

## Methods

### Ethical considerations

The study obtained ethical clearance from the North-West University Health Research Ethics Committee (approval number NWU-00312-20-A1). The National Health Laboratory Services manager for Academic Affairs and Research granted permission to access the data source. The head of the Limpopo Department of Health granted data access permission and use of the study centre name in publications. The North-West University Health Research Ethics Committee also granted a patient informed consent waiver since the data set was provided without patient names and other personal details (unique identification codes only). The data set was received in a password-protected Microsoft Excel® (Microsoft Corp., Redmond, Washington, United States) spreadsheet file and was stored on a password-protected computer. This manuscript does not report on the use of any animal or human data or tissue.

### Data collection

A retrospective, quantitative descriptive study design was adopted. The data were retrieved from the National Health Laboratory Service (NHLS) database, the national repository for all laboratory tests performed by South African public hospitals.^22^ No patient demographic and clinical data were extracted for the study. Extracted study variables include the ward name, specimen type (blood), patient unique identification codes, organism name, year of isolation, and antimicrobial susceptibility test outcome (intermediate, resistant, or sensitive).

The data represented all blood culture specimens performed at the 509-bed Mankweng Tertiary Hospital. There were 454 beds designated as either Level 1 (district) or Level 2 (regional), and 55 as Level 3 (tertiary services beds). The hospital is approximately 30 km east of Polokwane in the Capricorn District of Limpopo province, South Africa. Mankweng Tertiary Hospital is the primary hospital for the surrounding community (population of ~260 000 to 280 000). In addition, the hospital offers 24-h tertiary services and receives referrals from district and regional hospitals in Limpopo province.^23^

All ESKAPE pathogens isolated from bloodstream infection and their respective antibiotic susceptibility results from January 2014 to December 2019 were included in the study. The study period was chosen to correspond to available antimicrobial consumption data extracted from the Limpopo province Pharmaceutical Depot, which had previously been analysed and published. The data comprised all patients with positive blood cultures. Blood cultures are considered the ‘gold standard’ investigation for detecting micro-organisms in blood (bloodstream infections). The NHLS used different blood culture bottles to collect blood samples. Two blood culture bottles (aerobic and anaerobic) were used for adults, and one for paediatric patients.^24^ The samples are inverted 8–10 times.^24^ Blood culture samples were stored at 20 °C – 25 °C until the NHLS courier collected them from Mankweng Tertiary Hospital and transported to Pietersburg Tertiary Hospital NHLS laboratory for further processing (pathogen identification and AMR testing).

### Laboratory analyses

In the NHLS, blood culture bottles were sent to the microbiology laboratory for incubation in the automated blood culture system after inoculation with appropriate blood volumes.^24^ Once blood cultures became positive for growth, they were removed from the machine for manual subculture techniques and Gram staining following standard operating procedures.^24^ The laboratory used the VITEK^®^ 2 (Biomérieux, Marcy l’Étoile, France) automated systems for bacterial identification and antimicrobial susceptibility testing, except for colistin, where broth microdilution was used where indicated. The definitions of the WHO priority list for drug-pathogen combination ESKAPE bacteria were based on the definitions provided by the Centers for Disease Prevention and Control (Table 1).^25^ The VITEK^®^ 2 machine tests antimicrobial susceptibility using the broth microdilution method and 64-well plastic cards with 17–20 antimicrobial agents. The VITEK^®^ 2 AST-N255 cards were used for Gram-negative antimicrobial susceptibility testing and the AST-P603 cards for Gram-positive. The VITEK^®^ 2 antimicrobial test panel comprises amikacin, amoxicillin with clavulanic acid, ampicillin-amoxicillin, cefazolin-cephalexin, cefazolin, cefepime, cefotaxime-ceftriaxone, cefoxitin, ceftazidime, cefuroxime, cephalexin, chloramphenicol, ciprofloxacin, clindamycin, cloxacillin, ertapenem, erythromycin-azithromycin, fusidic acid, gentamicin, imipenem, linezolid, meropenem, moxifloxacin, mupirocin, nalidixic acid, nitrofurantoin, ofloxacin, penicillin, penicillin-ampicillin, piperacillin with tazobactam, sulphamethoxazole with trimethoprim, teicoplanin, tetracycline, tigecycline, tobramycin, and vancomycin. The NHLS employed the Clinical and Laboratory Standards Institute guidelines M100 for the corresponding year to interpret all antibiotic susceptibility results.^26^ The Clinical and Laboratory Standards Institute M39 guideline (Analysis and presentation of cumulative antimicrobial susceptibility test data) recommends empirical therapy with an antibiotic or antibiotics that demonstrated 80.0% to 90.0% susceptibility to the most relevant organisms associated with a specific infection.^27^

**TABLE 1:** Antimicrobial resistance phenotypes definition provided by the United States Centers for Disease Prevention and Control.

Antimicrobial resistance phenotype	Definition
VREF	*E. faecium* that has resistance to vancomycin
MRSA	*S. aureus* that has resistance to cloxacillin
VRSA	*S. aureus* that has intermediate or resistance to vancomycin
3GCR *K. pneumoniae*	*K. pneumoniae* that has intermediate or resistance to ceftriaxone
CR *K. pneumoniae*	*K. pneumoniae* that has resistance to meropenem
CRAB	*Acinetobacter* spp. that has either intermediate or resistance to meropenem
CRPA	*P. aeruginosa* that has either intermediate or resistance to meropenem
Multidrug resistance	*A. baumannii* or *P. aeruginosa* that has either intermediate or resistance to at least one drug in at least three of the following five categories: extended-spectrum cephalosporin (cefepime, ceftriaxone), fluoroquinolones (ciprofloxacin, levofloxacin), aminoglycosides (amikacin, gentamicin), carbapenems (imipenem, meropenem), and piperacillin with tazobactam
Reserve antibiotics	Colistin, linezolid and tigecycline

*E. faecium, Enterococcus faecium; S. aureus, Staphylococcus aureus; K. pneumoniae, Klebsiella pneumoniae; P. aeruginosa, Pseudomonas aeruginosa; A. baumannii, Acinetobacter baumannii;* 3GCR *K. pneumoniae,* third-generation cephalosporin-resistant *K. pneumoniae*; CR *K. pneumoniae*, carbapenem-resistant *K. pneumoniae*; CRAB, carbapenem-resistant *A. baumannii*; CRPA, carbapenem-resistant *P. aeruginosa*; MRSA, methicillin-resistant *S. aureus*; VREF, vancomycin-resistant *E. faecium*; VRSA, vancomycin-resistant *S. aureus*.

### Data analysis

Duplicates per patient were identified using unique patient identifiers and specimen collection dates (< 28 days). The data set was categorised into relevant variables and descriptive analysis was performed and presented as frequencies and proportions. Because of varying antibiotic susceptibility testing done by the NHLS over the years for some drug-pathogen combinations, *N*-values fluctuated. During data analysis, isolates that tested intermediate or resistant to antibiotics were classified as resistant strains according to the phenotypes (Table 1). The Mann-Kendall test with a *α*-value of 0.05 was used to determine the statistical significance of changes in pathogen incidence and drug-pathogen resistance based on frequencies. The analyses were performed using the *R*-statistics package version 1.1.5 (R Foundation for Statistical Computing, Vienna, Austria).^28^

## Results

The study extracted data on ESKAPE organism name, isolation ward and year, and AMR test outcomes, without patient sociodemographic or clinical data. The incidence of ESKAPE pathogens isolated from all positive blood cultures showed no significant variation between 2014 and 2019 (*p* = 0.133) (Table 2).

**TABLE 2:** Incidence of ESKAPE pathogens at a referral hospital in Limpopo, South Africa, 2014–2019.

Pathogen	2014		2015		2016		2017		2018		2019		Total		Trend *(p*-value)
	*n*	%	*n*	%	*n*	%	*n*	%	*n*	%	*n*	%	*n*	%	
*E. faecium*	0	0.0	7	5.2	26	26.8	25	21.9	26	20.0	23	12.8	107	14.3	0.339
*S. aureus*	39	42.9	37	27.6	25	25.8	45	39.5	47	36.2	75	41.7	268	35.9	0.132
*K. pneumoniae*	46	50.5	68	50.7	4	4.1	0	0.0	0	0.0	0	0.0	118	15.8	0.007
*A. baumannii*	0	0.0	16	11.9	31	32.0	37	32.5	48	36.9	68	37.8	200	26.8	0.009
*P. aeruginosa*	4	4.4	6	4.5	11	11.3	7	6.1	9	6.9	14	7.8	51	6.8	0.060
*Enterobacter* spp.	2	2.2	0	0.0	0	0.0	0	0.0	0	0.0	0	0.0	2	0.3	0.243

**Total**	**91**	**100.0**	**134**	**100.0**	**97**	**100.0**	**114**	**100.0**	**130**	**100.0**	**180**	**100.0**	**746**	**100.0**	**0.133**

ESKAPE, *Enterococcus faecium, Staphylococcus aureus, Klebsiella pneumoniae, Acinetobacter baumannii, Pseudomonas aeruginosa,* and *Enterobacter* spp.; *n*, sample.

Between 2014 and 2019, *S. aureus* (*n* = 268/746; 35.9%) was isolated most frequently, followed by *A. baumannii* (*n* = 200/746, 26.8%), and *K. pneumoniae* (*n* = 118/746; 15.8%) (Table 2). *Staphylococcus aureus* increased from 39 isolates in 2014 to 75 in 2019 (*p* = 0.132). *Acinetobacter baumannii* increased significantly from 11.9% (*n* = 16/134) in 2015 to 37.8% (*n* = 68/180) in 2019 (*p* = 0.009), while *K. pneumoniae* isolation declined from 46 in 2014 and 68 in 2015 to four isolates in 2016 (*p* = 0.007). No *K. pneumoniae* isolates were identified from 2017 to 2019.

Most isolates (*n* = 417/746; 55.9%) were from the neonatal ward and the intensive care unit (*n* = 60/746; 8.0%) (Table 3).

**TABLE 3:** Number of ESKAPE pathogens in the isolation ward at a referral hospital in Limpopo, South Africa: 2014–2019.

Ward name	Year							% contribution
	2014	2015	2016	2017	2018	2019	Total	
Neonatal ward	51	83	64	71	63	85	417	55.9
Paediatric ward	8	12	3	14	15	11	63	8.4
Intensive care unit	5	13	12	5	7	18	60	8.0
Male medical ward	8	9	5	6	5	20	53	7.1
Female medical ward	7	5	2	5	10	11	40	5.4
Burns unit	4	2	5	5	10	10	36	4.8
Casualty	1	1	2	4	6	9	23	3.1
Male surgical ward	2	1	-	1	3	7	14	1.9
Sub-acute ward	1	3	1	1	3	3	12	1.6
High care unit	-	3	1	-	3	2	9	1.2
Female surgical ward	-	-	1	-	3	1	5	0.7
Health information centre	2	1	-	1	-	-	4	0.5
Antenatal ward	-	1	-	-	-	2	3	0.4
Obstetric ward	2	-	-	-	-	-	2	0.3
Outpatient department	-	-	-	-	2	-	2	0.3
Maternity ward	-	-	-	1	-	-	1	0.1
Orthopaedic ward	-	-	-	-	-	1	1	0.1
Paediatric outpatient department	-	-	1	-	-	-	1	0.1

**Total**	**91**	**134**	**97**	**114**	**130**	**180**	**746**	**100.0**

ESKAPE, *Enterococcus faecium, Staphylococcus aureus, Klebsiella pneumoniae, Acinetobacter baumannii, Pseudomonas aeruginosa*, and *Enterobacter* spp.

**FIGURE 1:**
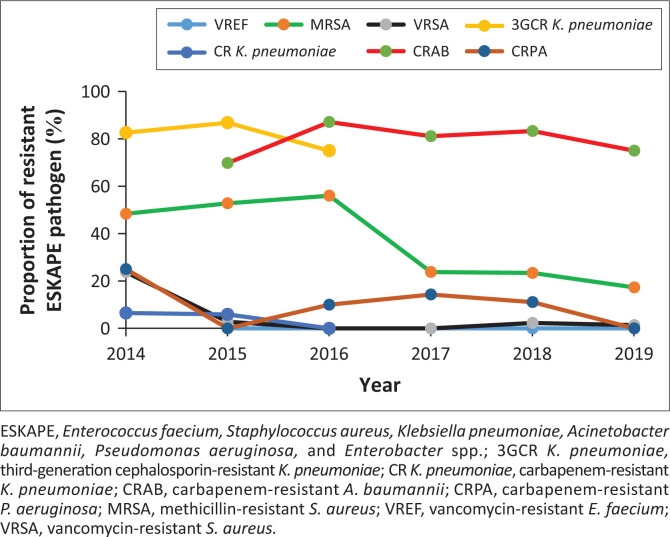
Drug-pathogen resistance of global priority tracer phenotypes at a referral hospital in Limpopo province, South Africa: 2014–2019.

Carbapenem-resistant *A. baumannii* increased significantly from 69.8% (*n* = 11/16) in 2015 to 75.0% (*n* = 51/68) in 2019 (*p* = 0.009) (Figure 1). The incidence of MRSA isolates decreased from 14 (56.0%, *n* = 25) in 2016 to 13 (17.3%, *n* = 75) in 2019 (*p* = 0.260). Vancomycin-resistant *S. aureus* was 4.7% (*n* = 12/256). In general, 3GCR *K. pneumoniae* incidence was 84.7% (*n* = 100/118), with no change in incidence rates from 82.6% (*n* = 38/46) in 2014 to 86.8% (*n* = 59/68) in 2015 and 75.0% (*n* = 3/4) in 2016 (*p* = 0.070), and carbapenem-resistant *K. pneumoniae* was 5.9% (*n* = 7/118) (*p* = 0.176). Carbapenem-resistant *P. aeruginosa* was 8.0% (*n* = 4/50).

*Klebsiella pneumoniae* (*n* = 0/36) was not resistant to colistin (see Online Supplementary Table 1). Colistin resistance was 1.1% (*n* = 2/190) in *A. baumannii* and 2.4% (*n* = 1/41) in *P. aeruginosa*. All samples for *E. faecium* (*n* = 0/100) and *S. aureus* (*n* = 0/193) were susceptible to tigecycline. Resistance to tigecycline was 10.7% (*n* = 20/187) for *A. baumannii* and 3.8% (*n* = 4/100) for *K. pneumoniae.* Linezolid resistance was found in 2.0% (*n* = 2/100) of *E. faecium* and 0.8% (*n* = 2/251) of *S. aureus* isolates.

Incidence rates for multidrug resistance between *A. baumannii* and cefepime, ciprofloxacin, gentamycin, meropenem, and piperacillin with tazobactam increased significantly from 2015 to 2019 (*p* = 0.008) (Online Supplementary Table 2). There was no change in the resistance rate for *P. aeruginosa* to amikacin (*p* = 0.242), cefepime (*p* = 0.060), ciprofloxacin (*p* = 0.176), meropenem (*p* = 0.817), and piperacillin with tazobactam (*p* = 0.159).

## Discussion

Key findings of our study are that ESKAPE pathogens blood culture isolates showed no significant variation between 2014 and 2019, and that *S. aureus, A. baumannii*, and *K. pneumoniae* were the ESKAPE pathogens commonly isolated in Mankweng Tertiary Hospital. Most of the ESKAPE isolates were from the neonatal ward, followed by the paediatric ward and the intensive care unit. There was an increase in CRAB, whereas MRSA decreased. Colistin resistance was less than 3.0% for Gram-negative pathogens *A. baumannii* and *P. aeruginosa*.

In our study, the blood culture isolates of the ESKAPE pathogens corresponded to the national trend in South Africa.^11^ Between 2018 and 2020, there was no significant variation in the proportion (ranging from 40% to 41%) of ESKAPE pathogens isolated from all public sector positive blood cultures in South Africa, despite an increase in the total number of blood cultures tested.^11^ A national neonatal public sector South African study from 2014 to 2019 also found that despite increased blood culture collection, the annual proportion of positive blood cultures remained unchanged, with an incidence risk of 6.0 per 1000 livebirths (95% confidence interval: 6.0–6.1).^13^ Antimicrobial use is a significant promoter of resistance, giving resistant bacteria a selection advantage over sensitive ones.^9^ The observed insignificant variation in the incidence of ESKAPE pathogens in our study and across the South African public sector^11^ could be attributed to the centralised medicines procurement contracts based on the national standard treatment guidelines and essential medicines lists.^11^ However, there is no definitive evidence to explain the stable incidence of ESKAPE pathogens in our study or across South Africa, therefore ecological studies are warranted. Despite the overall consistent ESKAPE pathogen incidence, our study showed variation in the incidence among individual ESKAPE pathogens, which could be attributed to antimicrobial selection pressure.

*Staphylococcus aureus, A. baumannii* and *K. pneumoniae* were the most frequently isolated ESKAPE pathogens in our study, similar to a countrywide public sector neonatal population study in South Africa between 2014 and 2019,^13^ as well as a South African national mixed-population report from 2020.^11^ Furthermore, our findings corroborate 2019 global^1,2^ and other single-centre trends from the Limpopo (2016) and KwaZulu-Natal (2011–2015) provinces in South Africa.^29,30^ The increased number of *S. aureus* isolates observed in our study is concerning, as global data show that *S. aureus* was the leading cause of fatal bloodstream infections, resulting in approximately 299 000 deaths and 30 million years of life lost in 2019.^1^ *Acinetobacter baumannii* was the sixth most isolated bloodstream pathogen globally in 2019.^1,2^

The high incidence of *A. baumannii* in this study is furthermore concerning since it is the leading cause of prolonged hospital stay,^2,10^ increasing the risk of hospital-acquired infections and escalating healthcare costs, and delaying patient access to care owing to bed shortages.^7,8,31^ Despite being one of the bloodstream infections isolated the most in our study, *K. pneumoniae* isolation decreased significantly during the study. This development was encouraging, since *K. pneumoniae* was the bloodstream pathogen isolated the most in South Africa between 2014 and 2019,^11,13^ and was responsible for higher mortality from bloodstream infections in sub-Saharan Africa than *S. aureus*.^1,4^ In South Africa in 2019, *S. aureus* infections were associated with 6000 deaths, *A. baumannii* with 6700, and *K. pneumoniae* with 3200.^12^ The increased incidence of multidrug-resistant Gram-negative *A. baumannii* in our study (hospital setting) could be attributed to inefficient infection prevention and control methods and limited antimicrobial stewardship.^13^

The higher ESKAPE pathogen incidence in the neonatal ward versus other wards could be attributed to the increased risk of neonatal sepsis resulting from risk factors such as prematurity, low birth weight, ruptured membranes, previous surgery, and intrapartum fever.^32^ These neonatal sepsis risk factors could lead to longer hospital stays, during which horizontal pathogen transmission could occur, particularly in overcrowded neonatal units with inadequate adherence to infection prevention and control measures.^13^ In addition, the variation in ward incidence could be attributed to differences in the type of healthcare services offered in neonatal, paediatric, and intensive care units, such as premature and low-weight neonates vulnerable to sepsis, vulnerable paediatric populations, and severely ill patients.^13,32^ The ward variation could also be attributed to differences in sampling practices across various hospital wards, where the causative pathogen(s) in bloodstream infections may go undiagnosed because minimal blood samples are collected.^13^ This assertion is substantiated by the findings of a 2018 study conducted at a tertiary hospital in Gauteng, South Africa, which showed that there was inconsistent adherence to blood culture collection guidelines, with over two-thirds (67.0%) of patients who were eligible for a blood culture not receiving one.^33^ Other single-centre neonatal and children’s studies from Gauteng, KwaZulu-Natal and the Western Cape in South Africa have previously identified ESKAPE pathogens as the dominant isolates of bloodstream infections between 2008 and 2018.^15,16,17,18,19^

The overall high and increasing incidence of CRAB in this study resembles the 80.0% for South Africa observed in 2020,^11^ and the neonatal population between 2014 and 2019 (*A. baumannii* isolates declined in susceptibility to meropenem [23.0% to 12.0%; *p* = 0.051]),^13^ which could drive the use of colistin since *A. baumannii* exhibited multidrug resistance in our study.^11,15^ In 2019, CRAB caused 50 000 to 100 000 AMR-related deaths globally,^1^ and was associated with the largest mortality impact of healthcare-acquired infections in low- and middle-income countries.^8^ There is a scarcity of data for *A. baumannii*-colistin susceptibility and/or resistance in low- and middle-income countries.^2^ *Acinetobacter baumannii* was susceptible to colistin in our study. In addition, this study demonstrated that Gram-negative *A. baumannii* and *P. aeruginosa* colistin resistance was below 3.0%, consistent with South African laboratory-based sentinel surveillance and routine data in 2016 to 2017.^11,15^ This could be attributed to the restricted use of colistin in the South African public sector.^11,34,35^

A 2020 South African national report showed *A. baumannii* tigecycline resistance at 15.0%,^11^ compared to 10.7% (*n* = 20/187) in our study. In South Africa, tigecycline is registered to treat complicated skin- and soft-tissue infections, intra-abdominal infections, and community-acquired bacterial pneumonia.^36^ As multidrug-resistant infections such as CRAB increase,^37^ clinicians may use high doses of tigecycline for unregistered indications such as sepsis,^37,38^ which can lead to secondary bacteraemia because of its bacteriostatic properties.^38^ According to a South African administrative report, clinicians were concerned with poor clinical outcomes when tigecycline was used alone.^11^ The emergence of tigecycline-resistant *A. baumannii* is complex as a result of multiple and multifaceted resistance mechanisms,^38^ necessitating comprehensive clinical evidence and research literature review before recommending alternate therapeutic choices. A medicine use evaluation study for tigecycline in our study setting is, therefore, recommended.

Methicillin-resistant *S. aureus* decreased in this study, similar to South African national surveillance data for 2016, 2017 and 2020,^11,14^ and previous single-centre studies conducted between 2011 and 2012 in the Gauteng and KwaZulu-Natal provinces of South Africa.^18,19^ This observation contradicts global MRSA data, which showed that MRSA increased by 14.0% from 21.0% in 2016 to 35.0% in 2020.^39^ The publication of the National Guidelines for the Prevention and Containment of Antimicrobial Resistance in South African Hospitals in 2017 may have contributed to the decrease in MRSA in our study, and in South Africa.^37^ However, more research on the progression of infections is needed to support this assertion.^40^ The incidence of MRSA remains a concern given the increased incidence of *S. aureus* in this study, as MRSA has the most significant effect on hospital stay (increasing the stay by 14 days compared to sensitive *S. aureus*) in low- and middle-income countries.^8^ In addition, the proportions of MRSA, together with 3GCR *Escherichia coli,* serve as global indicators for the 2030 Sustainable Development Goals to reduce AMR and require constant monitoring.^41^ *Staphylococcus aureus* was susceptible to vancomycin (susceptibility above 90.0%), making it an empirical choice, with tigecycline as a last resort.^42^

*Klebsiella pneumoniae* was the most isolated ESKAPE pathogen in South Africa in 2015 to 2017.^11^ Consequently, the absence of *K. pneumoniae* isolates during the last 3 years (2017 to 2019) of our study was important. The potential contributing factors were beyond the scope of our study and will require future research to provide practical implications. The resistance of 3GCR *K. pneumoniae* in this study is comparable to the South African report between 2015 and 2017,^11^ and other single-centre studies in South Africa from the Gauteng and Western Cape provinces of South Africa between 2008 and 2013.^18,43^ However, carbapenem-resistant *K. pneumoniae* in our study was low (< 10.0%), rendering carbapenems an effective alternative Watch antibiotic for the management of 3GCR *K. pneumoniae*, and Reserve antibiotics (colistin and tigecycline) as a last resort.^42^ The low carbapenem-resistant *K. pneumoniae* in our study could have accelerated the selection process, leading to extensive carbapenem use in 3GCR *K. pneumoniae* management, which could have increased CRAB growth.

Our study and single-centre studies conducted in the Western Cape province of South Africa between 2008 and 2018^21,43^ have yielded similar findings, not detecting vancomycin-resistant *E. faecium*. Nevertheless, the latest 2020 South African data indicated a vancomycin-resistant *E. faecium* incidence rate of 1.3%, which remains low.^11^ Based on this finding, vancomycin is considered an empirical choice for the treatment of bloodstream *E. faecium* infections. At the same time, Reserve antibiotics (linezolid and tigecycline) should only be used as a last resort.^44^ Compared to previous South African data from 2020, the incidence rate of carbapenem-resistant *P. aeruginosa* was low, at 33.0%.^11^ Furthermore, *P. aeruginosa* had low resistance to aminoglycosides and fluoroquinolones, making Access antibiotics (amikacin and gentamycin) and Watch antibiotics (ciprofloxacin and meropenem) empirical choices for bloodstream infections, with colistin as a last resort.^42^

### Limitations

Our study had the following limitations. Firstly, the study relied on a laboratory-based data set not linked to patient diagnoses and outcomes, limiting inferential analysis. Consequently, future patient-level pathogen-specific clinical studies are encouraged to describe morbidity and mortality risk factors associated with these infections, particularly in neonatal, paediatric, and intensive care units. Secondly, our study used routine laboratory data to report on phenotypic AMR of ESKAPE pathogens. We acknowledge that in an ideal setting, phenotypic and molecular surveillance AMR data are used together to classify bacterial isolates and describe the magnitude and mechanism of AMR. However, molecular AMR surveillance in South Africa is limited to the national referral laboratory; decentralisation to rural healthcare facilities is limited by lack of funding. Thirdly, the use of retrospective routine data does not allow for the determination of reliability and validity for certain observations. Fourthly, the requested annual blood sample data from the study data source were not provided. Consequently, due to data limitations, we could not determine the overall or individual incidence rate of bloodstream ESKAPE pathogens. Even though routine surveillance data are available in some low-resource settings, limited access to essential variables such as total blood cultures could present a challenge to the estimation of prevalence and surveillance of infectious diseases, as was the case in our study. Fifthly, our retrospective study design has the inherent limitation of low external study validity, and our findings could not be generalised to other settings. Lastly, other limitations included missing variables and selection bias as not all isolates were subjected to antibiotic susceptibility testing, limiting essential data analysis.

Future studies should consider confounding variables such as infectious disease diagnostic protocols, antibiotic stewardship programmes, and infection prevention and control measures to improve their contextualisation and interpretation.^41^

### Conclusion

This study has presented essential information on the incidence of ESKAPE pathogens and antibiotic resistance phenotypes included in the list of global priority drug-pathogen combinations in patients with bloodstream infections in Mankweng Tertiary Hospital in Limpopo province, South Africa. Therefore, it is essential to establish a reliable and timely surveillance system, implement and maintain infection prevention and control measures, and empirical alternative antibiotic treatment regimens. The present information could serve as a basis for the development of an institutional antibiogram that could be used together with institutional formulary and prescribing guidelines for optimal antibiotic use. The surveillance data could provide valuable drug selection information on appropriate empirical therapy before the availability of antibiotic sensitivity testing results for a specific patient.
